# Spatial Wavelet Analysis of Calcium Oscillations in Developing Neurons

**DOI:** 10.1371/journal.pone.0075986

**Published:** 2013-10-14

**Authors:** Federico Alessandro Ruffinatti, Alessandra Gilardino, Davide Lovisolo, Mario Ferraro

**Affiliations:** 1 Department of Life Sciences and Systems Biology, University of Turin, Turin, Italy; 2 Department of Physics, University of Turin, Turin, Italy; 3 NIS Interdepartmental Center, University of Turin, Turin, Italy; 4 Neuroscience Institute of Turin, University of Turin, Turin, Italy; University of Maribor, Slovenia

## Abstract

Calcium signals play a major role in the control of all key stages of neuronal development, and in particular in the growth and orientation of neuritic processes. These signals are characterized by high spatial compartmentalization, a property which has a strong relevance in the different roles of specific neuronal regions in information coding. In this context it is therefore important to understand the structural and functional basis of this spatial compartmentalization, and in particular whether the behavior at each compartment is merely a consequence of its specific geometry or the result of the spatial segregation of specific calcium influx/efflux mechanisms. Here we have developed a novel approach to separate geometrical from functional differences, regardless on the assumptions on the actual mechanisms involved in the generation of calcium signals. First, spatial indices are derived with a wavelet-theoretic approach which define a measure of the oscillations of cytosolic calcium concentration in specific regions of interests (ROIs) along a cell, in our case developing chick ciliary ganglion neurons. The resulting spatial profile demonstrates clearly that different ROIs along the neuron are characterized by specific patterns of calcium oscillations. Next we have investigated whether this inhomogeneity is due just to geometrical factors, namely the surface to volume ratio in the different subcompartments (e.g. soma vs. growth cone) or it depends on their specific biophysical properties. To this aim correlation functions are computed between the activity indices and the surface/volume ratio along the cell: the data thus obtained are validated by a statistical analysis on a dataset of 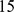 different cells. This analysis shows that whereas in the soma calcium dynamics is highly correlated to the surface/volume ratio, correlations drop in the growth cone-neurite region, suggesting that in this latter case the key factor is the expression of specific mechanisms controlling calcium influx/efflux.

## Introduction

The growth, orientation and specification of neuritic processes from developing neurons is a key event in the formation of the correct connectivity of the nervous system and is tightly regulated by a wide set of signalling mechanisms, among which complex spatiotemporal patterns of changes in cytosolic free calcium concentration, 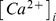, play a major role [1]. At the growth cone, the leading edge of a growing neurite, both spontaneous and agonist-induced changes in 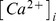 have been described (see e.g. [2], [3]): often they show an oscillatory behavior [4], [5], while somatic signals have in most cases a more sustained time course and oscillations, when present, are strongly attenuated [6]–[8].

Information coded by oscillations of 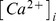 at the growth cone has been known to be relevant in determining its motility and morphology [2], [4], [5], [9], even if, at least in some instances, also signals generated at the soma, and propagated to neuritic/axonal compartment, have been reported to be involved in these processes [10]. The differences between signals at the soma and at thin peripheral compartments (such as filopodia of neurites and pre- and post-synaptic regions) have been ascribed, in most cases, to differences in the surface to volume ratio [11]–[13], but a contribution from spatial specificity in the calcium mobilizing mechanisms, based on the different distribution of channels (and/or transporters), may also be involved [14], [15].

We have tried to address this problem by simultaneously recording spontaneous calcium signalling activity from the soma, neurites and growth cones of E7 chick ciliary ganglion (CG) neurons in culture, and by generalizing a wavelet-based analytical approach described in a previous paper [16] to perform a spatial analysis of the oscillatory activity during a defined time interval in the different compartments of the neuron. The aim was to correlate the differences in oscillatory activity as a function of space with the estimated surface to volume ratio, in order to uncover any specificity of the different compartments in terms of calcium mobilizing mechanisms.

## Materials and Methods

### Cell Cultures

Chick ciliary ganglia (CG) were dissected from E7 embryos and maintained in a chemically defined N2 medium [17] as previously described [8]. Briefly, ganglia were both enzymatically (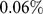 trypsin, in cation-free phosphate-buffered saline, for 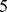 min at 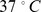) and mechanically dissociated and resuspended in N2 medium. Nearly 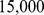 cells were plated in the middle area of glass coverslips coated with poly-D-lysine (PL; 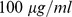) and laminin (LN; 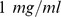) in N2 medium. If not otherwise specified, all chemicals were purchased from Sigma Chemical Co. (St. Louis, MO).

### Calcium Imaging

Calcium intracellular concentration was monitored using the ratiometric 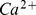 indicator dye Fura-2 acetoxymethylester (Fura-2AM, Molecular Probes, Inc.). Cells were loaded for 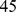 min at 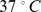 with 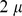
M Fura-2AM in N2 medium and subsequently washed in Tyrode Standard solution (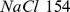 mM, 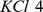 mM, 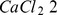 mM, 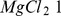 mM, 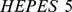 mM, glucose 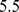 mM, pH 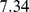 with 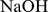). After dye loading cells were transferred to a perfusion chamber (Bioptechs, USA) and mounted on an inverted fluorescence microscope (Nikon TE-2000-S), a Xenon lamp illumination system and a CoolSNAP CCD camera (Roper Scientific/Photometrics, Germany). All experiments were performed at 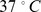. A gravity microperfusion system, regulated by electrovalves, was employed to keep the cells under a Tyrode solution laminar flow condition. Calcium measurements were performed exciting the probe for 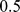 s alternatively at 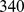 nm and 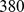 nm, with a dark interval of 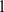 s (for a total sampling time of 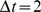 s), and recording the corresponding emission intensities (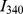 and 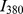) at 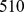 nm; the 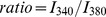 is an uncalibrated, quantitative measure of 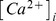. Images were visualized on a computer with the dedicated acquisition software Metafluor (Universal Imaging Corporation, PA). In order to obtain simultaneous recordings from the soma and the growth cone, experiments were performed after 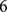
–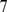 h of culture, when neurite extension was still limited and all the compartments could be observed by means of a 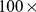 objective. One or at most two cells per dish could be recorded. Since at this short culture time cells could still be recovering from the dissociation procedure and some perturbation in the membrane could be expected, all cells were challenged with 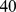 mM 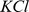 at the end of each experiment, in order to depolarize the membrane potential and elicit calcium influx through voltage-dependent calcium channels; non responsive cells were discarded.

### Wavelet Analysis

#### Preliminaries

In order to provide a quantitative evaluation of the spatial compartmentalization of oscillatory activity, we have developed an extension of the wavelet analysis tool we described in a previous paper [16]: the derivation is as follows.

**Figure 1:**
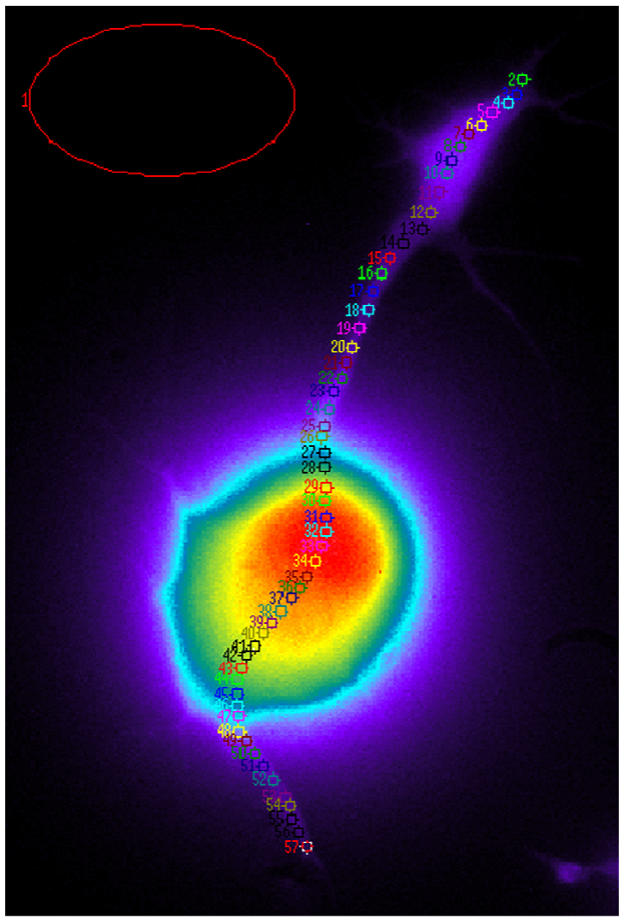
Image of a cell with ROIs superimposed to show space parametrization. The image was obtained from a cell loaded with the calcium indicator Fura-2 at 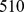 nm after excitation at 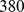 nm.

The starting point is to draw over the surface of each cell a large number of regions of interest (ROIs) as small as possible to cover the entire length of the cell as shown in Fig. 1. This procedure is repeated for each cell included into the database. All ROIs have the same shape and size, namely a circle with a radius of 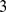 pixels, that correspond to about 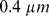: this size represents a good tradeoff between noise level and signal localization. The spatial position of the ROIs is parametrized, starting from the growth cone, by the discrete variable 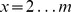 (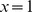 is always drawn outside the cell and used for the background subtraction); here 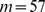. Each ROI 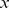 is associated to an oscillatory signal 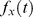 representing the local fluctuations of 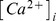.

Fourier transform represents a standard method to analyze these signals, but, since it is obtained via integration over time, it can provide information only on the frequencies making up the signal, and not on the time at which they occur. A more localized transform is needed to represent a signal in the time and frequency domain simultaneously, thus providing a better insight into the phenomena underlying the generation of the observed time courses, especially in the case of non stationary signals. A typical example is the windowed Fourier transform (or short time Fourier transform) where a moving window is shifted along the signal and the Fourier transform is computed just inside this window. Formally this can be obtained by multiplying the kernel of the Fourier transform by a window function 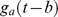: the parameter 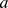 measures the width of the window, and the parameter 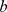 moves the window over the whole time domain. The resulting transform is the so called Gabor transform [18]. In this approach the value of 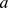, and so the width of the window, is fixed and this implies a trade-off between frequency and time resolution: small 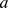 values give accurate information about the time course of the signal but they may lead to a coarse frequency representation, whereas large 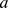 values provide high resolution of frequency and low time resolution, so that relevant events in the time course of the signal may be missed. This problem is solved by the so called multiscale analysis and, in particular, by wavelet transform, which takes the windowing procedure a step forward by making 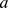 variable and replacing a single windowing function with a family of functions. A prototype function 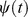 called “other wavelet”is selected and next the family 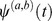 is constructed by means of translations and dilatations of the mother wavelet, corresponding to variations of 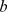 and 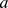 respectively.

For our purposes we chose Morlet wavelet as mother wavelet [19]:
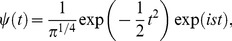
(1)where 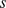 is a constant parameter (in this application 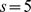). The family of functions originating from Eq. (1) and forming our wavelet basis is then
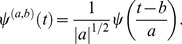
(2)


This set of functions defines the kernel of the wavelet transform 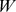:
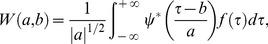
(3)where the asterisk denotes complex conjugation.

The critical point here is that 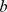 denotes the time at which 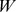 is computed while variable 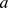 is related to the frequency through the relation 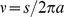; therefore 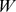 can be explicitly expressed as a function of both time 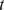 and frequency 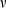.

The literature on wavelets and their applications is huge: a clear historical introduction can be found in [18] and an in depth treatment is provided, for instance, by [10] and [21].

In this application then, for each ROI 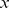, the wavelet transform of 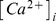 time course is computed:
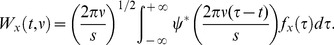
(4)


Here 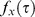 represent the time course of the concentration 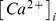 recorded from the 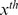 ROI, 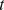 is the time and 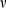 is the frequency variable. Thus in conclusion
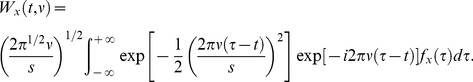
(5)


The modulus 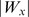 of the wavelet transform can be used, in a variety of ways, to describe the time evolution of the activity at each ROI. An instance is the so called energy density 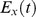 (see [22]),
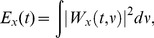
(6)in which contributions from all frequencies are integrated to provide a function of time. The time-averaged energy density of the signal within a time interval 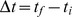 is
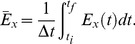
(7)


Note that
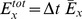
(8)can be indeed regarded as the total energy of the signal recorded from the 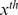 ROI during 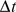 time interval, thus providing a suitable representation of energy as a function of space. However, in most cases of interest the main contributions to energy density, at every time point, are concentrated around a few maxima [16], that correspond to the most relevant events in the signal, such as sharp peaks or oscillatory bursts; in turn these events are characterized by the occurrence of high frequency components. Therefore relevant changes in the signal can be highlighted by defining an activity index taking into account only the maxima of 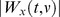. This can be done, for instance, by summing the contributions of the maxima of 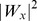 weighted by the frequencies [16], namely
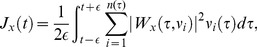
(9)where 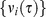 is exactly the set of local maxima of 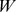 along the 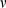 axis, at time 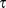. Since the number of these maxima changes in time, parameter 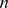 is expressed as a function of 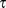. Integration simply serves to smoothen the index, by avoiding abrupt variations due to discontinuities of frequency paths.

For future use we define 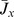, the time average of 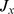, as
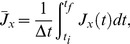
(10)where 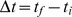 is the temporal window of observation. This new index provides for every 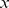 value an integral measure of oscillatory activity.

### Spatial Trends of Activity

Dissociated CG neurons in short term (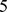
–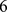 hours) culture actively protrude (and in some cases retract) one or more neurites [23]. During this process, a subpopulation (about 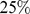 out of 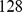 cells) showed spontaneous oscillations in 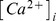, in the absence of any exogenous signal. This behavior was in general more evident at the growth cones, while the proximal neurites and the soma were less involved.

**Figure 2:**
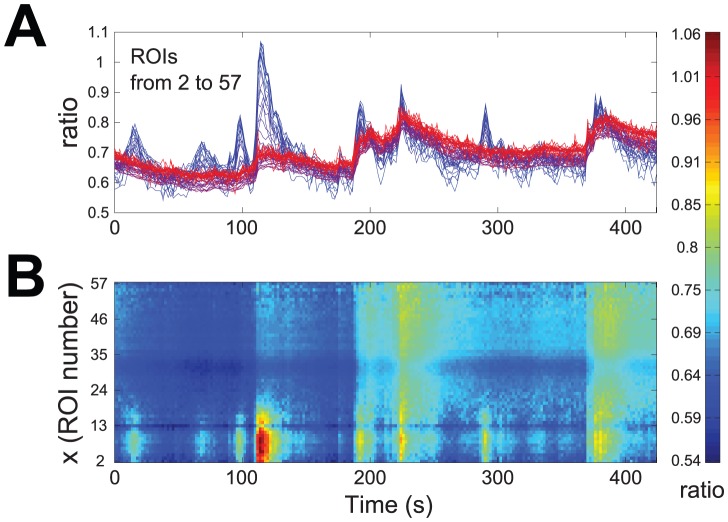
Time courses of 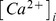 recorded from all ROIs of the cell of Fig. 1. A: time courses superimposed according to a color gradient from blue (growth cone: low 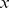 values) to red (soma: high 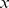 values). B: two-dimensional map of the same data. The horizontal and vertical axes are, respectively, time (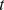) and space (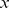) coordinates, while the concentration is coded by colors, from blue (low 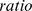 values) to red (high 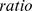 values).

**Figure 3:**
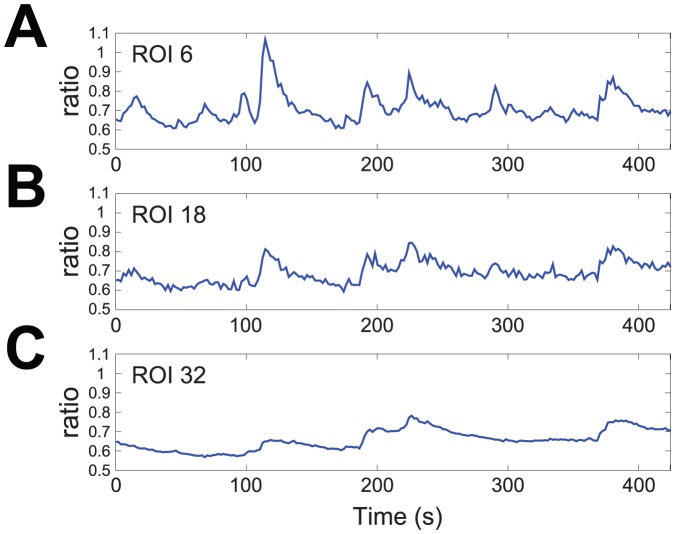
Three prototypical traces, extracted from the plots of **Fig. 2A**. Growth cone (A: ROI 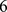), neuritic shaft (B: ROI 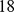) and soma (C: ROI 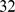).

Figure 2A shows the time courses of the change in 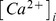 recorded from all ROIs of the neuron shown in Fig. 1 and superimposed according to a color gradient from blue (corresponding to the growth cone: low 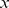 values) to red (soma: high 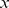 values). The same data are presented in Fig. 2B via a two-dimensional map: the horizontal and vertical axes are, respectively, time (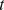) and space (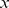) coordinates, while the concentration is coded by colors, from blue (low 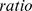 values) to red (high 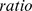 values). Even though traces from all ROIs exhibit an oscillatory behavior, it appears to depend on the spatial index 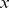, as can be seen from Fig. 2B. This point is highlighted in Fig. 3 by considering three traces extracted from the recordings of Fig. 2A, respectively from the growth cone (A: ROI 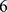), the neuritic shaft (B: ROI 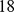) and the soma (C: ROI 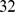). It is evident that at the growth cone the oscillations in 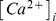 are of greater amplitude and their rising and decay phases have more rapid kinetics than in the other two compartments: a quantitative evaluation of the differences in the oscillatory activity of the three traces of Fig. 3 can be provided by plotting the related scaleograms obtained by wavelet transformation (see Fig. S1), and information on spatiotemporal localization of the most relevant oscillatory events can be extracted by deriving from each scaleogram appropriate measures, such as 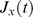.

**Figure 4:**
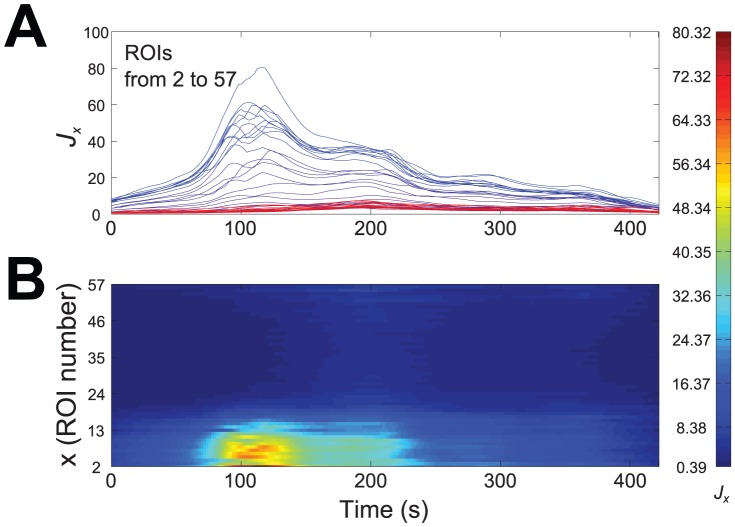
Activity index 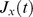 computed for all ROIs. A: trends of 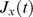 for all ROIs, superimposed according to a color gradient from blue (growth cone) to red (soma). B: two-dimensional map of 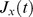. The horizontal and vertical axes are, respectively, time (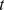) and space (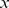) coordinates, while values of 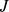 are coded by colors, from blue (low) to red (high).

**Figure 5:**
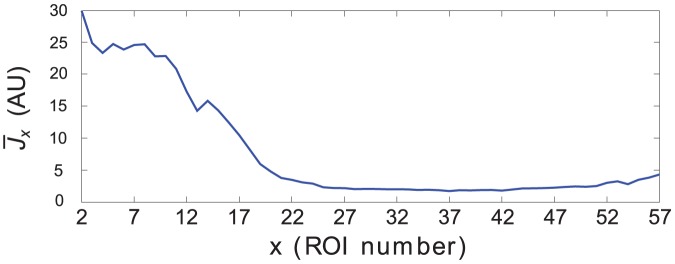
Spatial trend of time-averaged activity index. Graph of 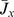 as a function of ROI position, from the growth cone to the soma.

Figure 4A shows the trend of 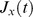 for all ROIs (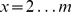), superimposed according to a color gradient from blue (growth cone) to red (soma). In Fig. 4B the same data are presented in a color coded 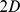 map: the horizontal and vertical axes are, respectively, time 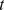 and space 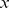 coordinates, while the 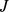 value is coded by colors, from blue (low values) to red (high values). The map of 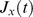 demonstrates that this index can capture the most relevant features of oscillatory activity: it shows clearly that such activity is confined in a well defined spatiotemporal domain of about 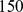 s in time duration and spatially restricted at the ROIs in the growth cone. A spatial representation of activity is provided by time-averaged index 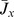, whose graph is plotted in Fig. 5 and shows that indeed oscillatory activity is large in the growth cone (up to 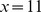) and then declines sharply along the neurite, while in the soma it is small and nearly uniform.

These results do not depend on the particular index used here. Similar maps and plots can be derived via the energy density 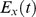 and its time average 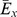 (see Fig. S2, S3).

### Exploring the Influence of Geometry

#### Preliminaries

The variation of 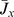 along the neuron, as shown in Fig. 5, clearly points to a specificity of different neuronal subcompartments as activity decreases from the cone to the soma. The question is then whether these differences are solely ascribable to scale factors in the geometry of the cells.

The idea that scale factors can, and indeed do, affect functions of organisms dates back to Galileo [24] and scale laws have been found to regulate a variety of biological mechanisms (see for instance [25] and, for more recent references, [26] and [27]). In particular surface to volume ratio has been long known to be important for the life cycle of the cell [28], [29], but also for other functional processes such as information coding at the growth cone of the extending neurites (see e.g. [11]).

To investigate further this point we computed the correlation along the cell between 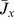 (indexing the local oscillatory activity) and 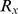, the surface to volume ratio in a given ROI.

It is a very general rule [25] that volume and surface can always be expressed as powers of some characteristic length 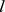 so that 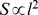 and 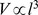: then the surface to volume ratio 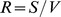 scales as 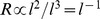. In our case the characteristic length 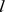 is the thickness of the cytosolic region under each ROI. Assume all ROIs to be small circles of the same area, say 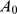, then the underlying volume of a single ROI turns out to be a cylinder: this is a first order approximation but, as shown later, it will not affect the thrust of our analysis. The sum of the top and the bottom area of this cylinder is coincident with the double area of each ROI and, since we consider that influx/efflux of calcium occurs prevalently at the plasmamembrane, 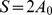. On the other hand, the volume is 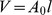 for every single cylinder, then 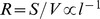 also holds in our case. Clearly the thickness of the cell is a function of 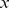, as it varies from ROI to ROI: thus the characteristic length will be labeled as 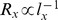.

### Surface to Volume Ratio Assessment

We have developed a simple approach to estimate cell thickness 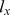 under the surface of the single ROI as a function of space 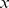, using the same ROI parametrization already employed for the computation of 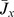.

The method consists of two steps: first we need to identify one or more time points at which the 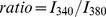 is the same for each ROI of the cell, i.e. at which 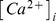 is quite homogeneous all over the cell (in our case see Fig. 2: 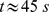); then, under this condition, we can plot, as a function of 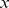, the fluorescence intensity recorded just from one of the two Fura-2 excitation wavelengths as a reliable estimation of the volume underlying the surface of each ROI. In effect, if 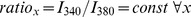 over the cell, then 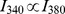 and both intensities are solely functions of the volume of cytosol under the 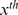 ROI. Because of the low calcium concentration in basal conditions, typically the 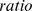 is 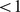 and then 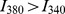: for this reason, although 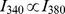 for each ROI, fluorescence intensity emission after excitation at 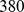 nm is often a more suitable measure than 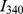.

**Figure 6:**
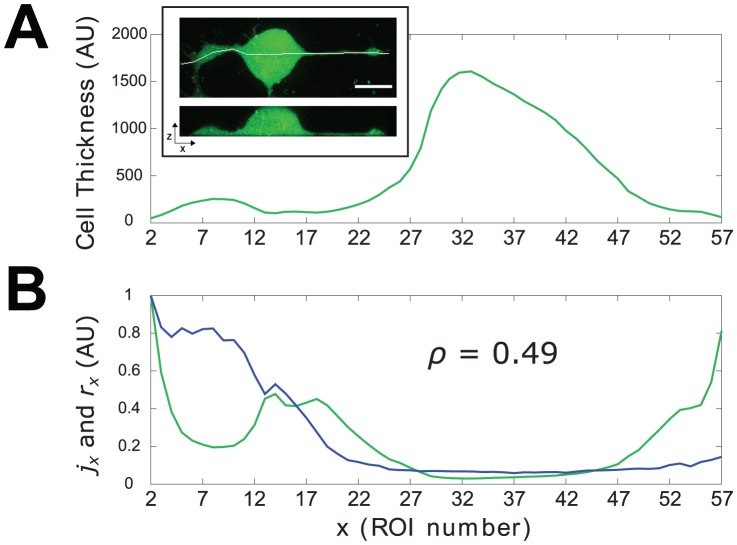
Cellular thickness and surface to volume ratio compared with oscillatory activity in space. A: profile of the thickness of the cell as derived with the procedure outlined in “urface to Volume Ratio Assessment” subsection. Inset: confocal image of a CG neuron cultured for 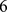 h in N2 medium and loaded for 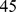 min with the fluorescent probe Fluo4-AM. Upper panel: 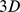 image reconstructed from XYZ scan; lower panel: virtual section along the horizontal white line in the upper panel. B: traces of the normalized activity index 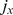 (blue trace) and surface to volume ratio 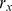 (green trace) along the cell. 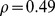 is the value of the correlation coefficient computed over the entire cell (global 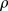).

In conclusion, we can assume 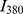 to be proportional to the cytosolic volume under each ROI and, being the area of all ROIs identical, the single wavelength plot gives an estimation of cytosolic thickness 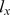 as a function of space, while its reciprocal 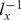, for the same reasons, is proportional to the cellular local surface to volume ratio 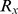. Fig. 6A shows the result for the cell of Fig. 1.

Note that by adopting this procedure we have done some assumptions that deserve to be addressed explicitly:

- Fura-2 is a cytosolic probe: in our work the term *volume* (as well as *thickness*) refers only to the compartment actually occupied by the probe, that is the cytosol, net of any possible organelle;
- all ROIs are drawn small enough (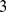 pixels 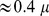
m of radius) to be coincident with the underlying portion of cellular membrane: in this work the term *surface* refers only to the plasmalemma patches delimiting, on the upper and lower side, those cylindrical volumes defined by each ROI;
- we do not keep into account the possible contribution of subcellular organelles to the changes in 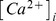. When addressing the problem of the dependence of changes in 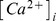 from the surface/volume ratio, it is usually implied that the surface is that of the plasmamembrane, and that changes in 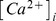 are dependent on influx form the extracellular medium (see e.g. [11], [14]). In our case, it has been already shown [6], [8] that release from the endoplasmic reticulum plays only a minor role in the signals elicited by a typical agonist such as basic Fibroblast Growth Factor (FGF-2); moreover, spontaneous calcium signals, both at the growth cone and at the soma, are completely suppressed in a calcium free extracellular solution (unpublished data). Therefore, we will assume that the relevant mechanism is calcium influx and the relevant parameter is the ratio between the cytosolic volume and the plasmamembrane surface, even if some contribution from calcium release cannot be excluded in principle.

In conclusion, even though Fura-2 is not an actual volumetric probe, our approach allows, at least on a first approximation, a reliable estimate of cellular thickness: its validity can be tested by comparing the plot in Fig. 6A with the information that can be obtained by confocal imaging. The inset of Fig. 6A shows a representative image of a cross section of a neuron cultured in the same experimental conditions and loaded with the cytosolic calcium indicator Fluo-4; the similarity of the two profiles is evident.

If on one hand the points mentioned above represent somehow the limits of the approach described here, on the other hand it turns to be a very practical approach because it allows to quantify calcium cytosolic concentration and cellular volume at once and with a single probe loading.

## Results

The influence of geometry on the oscillatory changes in 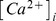 should be mirrored by the degree of agreement between 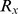 and 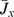. To asses such agreement quantitatively it is useful to resort to adimensional normalized indices derived from 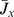 and 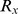, respectively:
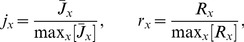
(11)where the 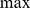 operator runs over all ROIs.

Traces of 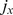 and 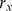, presented in Fig. 6B, show that in several ROIs the level of correspondence between surface to volume ratio and oscillatory activity is quite low. The global correspondence can be quantified by computing the correlation coefficient (Pearson product-moment) between 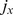 and 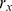 defined as
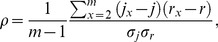
(12)where 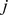, 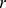 are the means of 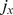 and 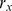 averaged on all ROIs and 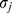, 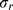 are the standard deviations. In the case of the cell used in our example 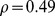. The use of 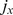 and 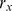 helps in understanding the plot, and hence the relation between activity and surface to volume ratio, but it is straightforward to show that the results of the correlations do not depend on the normalization and the same values for correlation coefficients would have been obtained by using 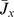 and 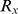.

To ensure that this result is not due to the particular index used here, we have computed the correlation between 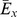, as defined in (7), and 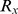 obtaining a similar value, 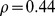.

For statistical purposes correlation coefficients 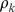 have been calculated between normalized indices 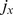 and ratios 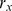 of 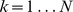 cells, where 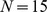 is the size of our sample. The mean correlation of our sample is 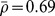 and the standard error of the mean (SEM) 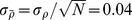. The relative standard error is 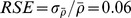 and this small value indicates that our estimate of 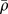 is quite reliable and our sample is large enough.

More relevant for our purposes is the fact that the agreement between the trends of 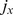 and 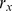 varies along the neuron (see Fig. 6B). To investigate this point we have considered two compartments of the cell: the first comprises the cone and the neurite and the second the soma of the neuron. We have then computed, again for each of the 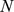 cells under consideration, two separate correlation coefficients 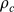 and 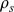 for the two compartments (growth cone/neurite complex and soma respectively): let 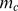, and 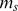 be the number of ROIs belonging to each compartment (in general being 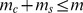) and let 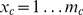, 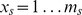 index the ROIs for each compartment, then
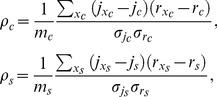
(13)where means and standard deviations are computed for each compartment of the cell. For the same cell as above, 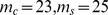 and 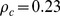, 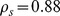. This first result shows that the effects of surface to volume ratio on the activity differ sharply in different subcompartments of the cell: in the soma the activity appears to follow closely the trend of 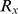, whereas they are quite independent in the growth cone-neurite complex. In particular consider the differences 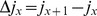 and 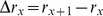: from Fig. 6B it is apparent that 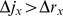 at the growth cone, whereas along the neurite 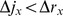 showing that the oscillatory activity in the growth cone is more sustained than what one could have expected just on the basis of the local surface to volume ratio. The change of sign in the previous inequality occurs at a point between the end of the cone and the beginning of the neurite and this is not an isolated case, but it represents a recurring scenario in the cells we have examined: growth cone likely exhibits a wide set of calcium mobilizing mechanisms that allows it to have great amplitude signals in spite of the fact that here the volume is greater than in the neurite, that seems instead to behave like a passive element.

Statistics of 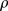, 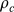, 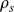, computed on the three data samples 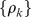, 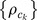, 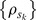, clearly show the differences between correlations in the two compartments:
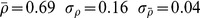
(14)

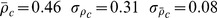
(15)

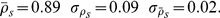
(16)


Notice once again that the results obtained with 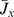 do not depend on its particular functional form, but they are consistent with other measures of activity. To enable a comparison we also report the statistics of the correlation coefficients computed using 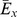 instead of 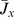 for the measure of the oscillatory activity:
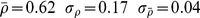
(17)

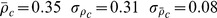
(18)

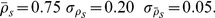
(19)


We can conclude that correlations are robust with respect to the choice of the activity index, but 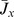 provides a slightly better result in terms of discrimination between somatic and neuritic compartment because 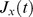, and hence 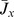, amplify the contribution of the high frequency components of the signal, thus highlighting the differences in oscillatory behavior.

Next the statistical analysis of these results is presented.

### Statistical Analysis

A bootstrap procedure was applied to the three sets of data (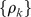, 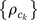, 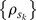) to draw sampling distributions and related confidence intervals. For each data set 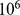 bootstrap samples of size 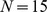 were generated and the histograms representing the distributions of their means were derived and plotted over 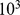 bins spanning from 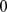 to 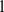.

**Figure 7:**
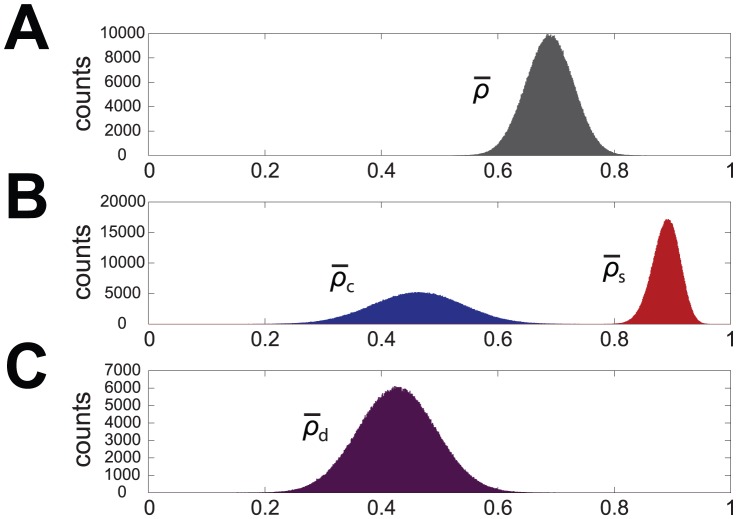
Bootstrap distributions, obtained by means of 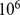
 **iterations and bootstrap samples of size** 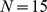 **.** A: bootstrap distribution of 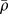. B: bootstrap distributions of 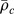 (blue bars) and 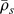 (red bars). C: bootstrap distribution of 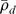.

The mean and the standard deviation of the so obtained bootstrap distribution should estimate respectively the mean and the standard error of the mean (SEM) of the original data and indeed the bootstrap distribution of 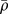 has mean and standard deviation that agree very well with mean and SEM of the sample 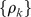. This distribution is shown in Fig. 7A and similarly Fig. 7B presents the bootstrap distributions of 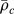 (blue bars) and 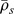 (red bars), respectively; even in this case the agreement between empirical and bootstrap statistics is very high.

Fig. 7B also shows a clear difference between the standard deviation of the two distributions: in particular the inequality 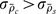 suggests that the main contribution to the standard deviation of the global correlation comes from the growth cone/neuritic compartment while the soma shows a more homogeneous behavior. In turn this may reflect the fact that while at the soma the surface to volume ratio is the most determinant feature influencing 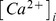 dynamics, neuritic shaft and growth cone markedly can express a large variety of behaviors independent from 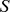/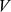 ratio, and instead dependent on other biophysical properties of the cell.

The statistical significance of the difference between the mean correlations in the two regions was estimated by considering the new statistical variable 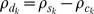 with 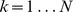. By this way we also keep into account the pairing of the two samples 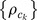, 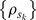. The resulting bootstrap distribution of 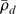 is shown in Fig. 7C, and the related 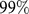 confidence interval is 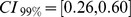: the lower bound of 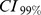 is well above zero and then we can conclude that 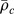 and 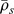 differ significantly with a significance level of 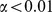. This significance level has also been confirmed by the result of a Wilcoxon signed-rank test.

It should be noted that the first order approximation we adopted may underestimate the ratio 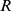 at ROIs whose diameter is comparable to the linear dimension of the cellular structure, as it may happen in the neurite. However, with higher order approximations one should expect for the cone-neurite compartment 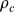 values lower than found here. This point has been verified by using on a cell a second order approximation, that takes membrane curvature into account. Thus first order approach produces the most conservative estimate of the difference between 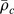 and 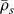 and yet this difference results to be statistically significant. The computational cost of using non-linear approximations is then not justified in this case.

### Computational Considerations

Wavelets are nowadays a standard tool in the analysis of many different types of signals, and many related software packages are readily available. However, we have chosen to further develop an original software tool, specifically tailored to our aims, called KYM ver.0.5, an early version of which has been first presented in [16].

KYM is fully compatible with both GNU Octave and MATLAB environment and it has been tested with the latest releases of these two environments (Octave 3.6.2 and MATLAB 8 (R2012b)).

Wavelet transform computation is here implemented as a product in the Fourier transformed domain and this ensures a relatively low computational complexity of 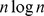 order, 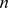 being the length of the signals. A standard code for this algorithm can be found, for instance, in WaveLab850 (http://www-stat.stanford.edu/wavelab/). Peak detection uses a technique that is based on image dilation (see, for instance, localMaximum.m m-file by Yonathan Nativ, http://www.mathworks.com/matlabcentral/fileexchange/authors/26510/). The rest of the code has been written and developed ad hoc to perform the analysis presented here.

KYM ver.0.5 can be freely downloaded from the well-established public-domain repository SourceForge (http://sourceforge.net/projects/kym/).

To our knowledge this is the first open source tool specifically dedicated to the analysis of the time course of cellular calcium signals and, more generally, of oscillatory signals recorded by means of fluorescent dyes from biological systems.

## Discussion

Calcium signalling in cells, and particularly in neurons, is characterized by high spatial compartmentalization, and this property has a strong relevance in the different roles of specific neuronal regions in information coding: see e.g. the contribution of dendritic subcompartments (spines, dendritic shaft, proximal vs. apical dendrites) as compared to somatic signals [12], [30]. This is true not only in mature neurons, but also during development: the involvement of calcium signals in the control of elongation, orientation and arrest of the growth cone of extending neurites, leading to the establishment of neuronal networks [4], [5], [9], [11], [31], is one relevant example. These signals usually have a marked oscillatory pattern, and their frequency has been shown to affect neurite growth.

A relevant issue in this context is the understanding of the structural and functional basis of this spatial compartmentalization: is the peculiar oscillatory behavior at the growth cone a passive consequence of the specific geometry of this subcellular region or a specificity of the spatial localization of calcium influx/efflux mechanisms can be evidenced? Davenport et al. [11], in a pioneering paper on the sensing machinery at the filopodia of the growth cone, ascribed it to “urely physical dimensions”, mainly the surface to volume ratio; while this parameter is without doubt relevant, others, mainly the inhomogeneity of distribution of membrane proteins, such as channels and transporters, may explain the specificity of these signals during the development and stabilization of neuronal networks.

The specific contribution of subcellular compartments to calcium signalling has been addressed mainly through two approaches: I) experimental manipulation of the signal by means of local application of specific agonists and blockers of different channel types (see e.g. [9]) and II) modeling of the influx/efflux mechanisms and of the buffering properties of the cytosol (see e.g. [32], [33]). Here we have developed a new and different approach, independent on assumptions on the actual mechanisms involved in the spatial specificity, that can be used as a predictive tool to separate functional differences from geometrical ones.

First, we defined a spatiotemporal index 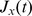 whose time average 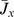 provides a spatial measure of the oscillatory events in calcium concentration, and shows that different neuronal subcomparments are characterized by different oscillatory activities. How this inhomogeneity can be attributed just to geometrical factors, namely the surface to volume ratio, has been investigated by computing correlations between the normalized versions of 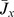 and 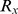, the local surface to volume ratio. The results show that the oscillatory activity is specifically localized at the growth cone, and its spatial distribution along the whole neuron is poorly related to the surface/volume ratio: along the neuritic shaft, where the ratio is high, activity drops. A statistical analysis on a dataset of 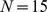 cells, has confirmed that in the soma calcium dynamics are correlated to the surface/volume ratio, whereas correlation drops in the growth cone-neurite complex, suggesting a spatial segregation of the properties of the growth cone and of its sensing machinery.

Apparently, signals at the growth cone maintain their local nature and are not fully propagated to the other compartments. Other works (see e.g. [34] for cerebellar granule cells) have shown that calcium signals elicited at the growth cone by an extracellular cue can propagate to the soma by means of a mechanism based on calcium-induced calcium release (CICR). This does not seem to be true in our case. As discussed above, calcium release does not play a major role in our experimental model.

Evidence that the oscillatory pattern of calcium signals is dependent on spatial specificity of membrane properties (and not exclusively on geometrical parameters) has been given by [8], for signals activated by a neurotrophic factor in the same experimental model: the growth cones of two neurites of the same neuron, of comparable morphology, showed markedly different oscillatory behaviors. A similar observation can also be found in Fig. 6 of the present paper, in which the second neurite does not show a marked oscillatory activity in spite of its high surface to volume ratio.

It must be remarked that our results refer uniquely to the spontaneous activity of the somatic, neuritic and growth cone compartments; while these signals have been reported to be relevant in the decisions the neuron and its processes have to take (see e.g. [31]), their behavior is strongly affected by a wide set of extracellular signals and their modulation of calcium signalling at the different subcompartments [1], [8]. In this regard, it should be noted that the method discussed here is not restricted to the present application, but it can be used to analyze spatial specificity of calcium signals in a variety of cases, such as agonist-elicited responses in a wide set of cellular models.

## Supporting Information

Figure S1
**Scaleograms: maps of wavelet transform modulus (** 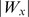 **) computed for the same three traces shown in** **Fig. 3**. Growth cone (A: ROI 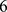), neuritic shaft (B: ROI 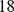) and soma (C: ROI 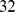). The horizontal and vertical axes are, respectively, time (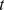) and frequency (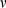) coordinates, while 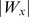 amplitude values are coded by colors, from blue (low) to red (high). High frequencies (representing the most rapid events, i.e. the sharpest peaks) are confined into the growth cone and they disappear moving toward the soma. The middle range frequencies are still present at the neurite, but at the soma only a weak component of the low frequency range has survived.(EPS)

Figure S2
**Energy density computed for all ROIs.** A: trends of 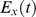 for all ROIs, superimposed according to a color gradient from blue (growth cone) to red (soma). B: two-dimensional map of 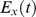. The horizontal and vertical axes are, respectively, time (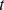) and space (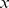) coordinates, while values of 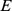 are coded by colors, from blue (low) to red (high).(EPS)

Figure S3
**Spatial trend of time-averaged energy density.** Graph of 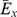 as a function of ROI position, from the growth cone to the soma.(EPS)
